# Dataset on droplet spreading and rebound behavior of water and viscous water-glycerol mixtures on superhydrophobic surfaces with laser-made channels

**DOI:** 10.1016/j.dib.2025.111697

**Published:** 2025-05-23

**Authors:** Matic Može, Samo Jereb, Robert Lovšin, Jure Berce, Matevž Zupančič, Iztok Golobič

**Affiliations:** Faculty of Mechanical Engineering, University of Ljubljana, Aškerčeva cesta 6, SI-1000 Ljubljana, Slovenia

**Keywords:** Droplet impact, Droplet-surface interaction, Restitution coefficient, Maximum spreading factor, Nonwetting surfaces, Laser-textured surfaces, Superhydrophobic surfaces, Self-cleaning surfaces

## Abstract

Droplet impact, spreading and rebound was investigated experimentally on superhydrophobic laser-textured surfaces, yielding a dataset of 1498 datapoints. Data was collected on twelve types of surfaces with square grids of laser-made channels of various spacings (from 50 µm to 800 µm), with two groups of six surfaces possessing either deep or shallow laser-made channels. Droplet impact tests were performed with water and viscous water-glycerol mixtures with viscosity values up to 160 mPa·s and droplet impact behavior was images with a high-speed camera at 5000 fps. Maximum spreading factor, contact time, droplet rebound efficiency, and maximum lamella velocity were extracted from the videos using software image processing. Moreover, information on droplet diameter, velocity, density, surface tension, dynamic viscosity, Weber number, and Reynolds number are provided. A supplementary dataset includes the same quantitative information for droplet impacts on a smooth, hydrophobic surface, resembling the surface between the laser-made channels on other superhydrophobic surfaces (125 additional datapoints). Furthermore, scanning electron microscopy images of the surfaces are provided alongside the measurements of static and dynamic contact angles with water and water-glycerol mixtures.

The data may be useful for fields like wettability studies, surface engineering, and anti-icing research. It can help validate theoretical and numerical models of droplet spreading, retracting, and rebounding from poorly wettable surfaces, optimize superhydrophobic surfaces for applications such as self-cleaning and drag reduction, and contribute to machine learning models predicting droplet behavior. The data is particularly relevant for designing anti-icing surfaces by minimizing contact time and maximizing the restitution coefficient. Additionally, it supports applications in 3D printing, coating technologies, and inkjet printing by providing data on viscous liquid impacts on poorly wettable surfaces.

Specifications Table

**Table:** 

Subject	Engineering & Materials science
Specific subject area	Wetting phenomena
Type of data	Processed data in .csv format, with supplementary processed data in .csv and .tif format.
Data collection	Droplet impact, spreading, and rebound on surfaces with different surface properties was captured using backlit imaging with a high-speed optical camera. The quantitative data was extracted from the captured footage via image processing with custom-developed algorithms in MathWorks MATLAB software environment.
Data source location	The data were collected at the Faculty of Mechanical Engineering, University of Ljubljana, Aškerčeva cesta 6, 1000 Ljubljana, Slovenia.
Data accessibility	Repository name: Mendeley Data Data identification number: http://dx.doi.org/10.17632/wsh8rxwd38.1 Direct URL to data: https://data.mendeley.com/datasets/wsh8rxwd38/1
Related research article	None.

## 1 Value of the Data

The following key possible uses of the dataset and the supplementary information are pointed out:

• A comprehensive dataset consists of 1498 droplet impact experiments using water and viscous water-glycerol mixtures on superhydrophobic laser-structured aluminum samples. This may aid the development of new physical models on droplet spreading with a focus on specific correlations for viscous liquids. Moreover, the stepwise variation of surface geometry supports modelling of surface properties as a function of spatial distribution of surface features.
• The data may be used for advancing the fundamental understanding of droplet-surface interactions, including droplet spreading, contact time, and rebound dynamics. It can be used to validate theoretical models and improve numerical simulations, particularly for surfaces with various surface patterns of modified areas.
• Additionally, the dataset allows for analyzing the role of viscosity in droplet dynamics, extending its relevance beyond water to more complex fluids such as biofluids, lubricants, and inks. Understanding these interactions is essential for optimizing surface designs in various engineering applications, including spray cooling, inkjet printing, and liquid-repellent coatings for food and pharmaceutical industry.
• The dataset can also be used for optimization of superhydrophobic surface designs for self-cleaning, anti-fouling, and anti-icing applications. By analyzing how different grid spacings and channel depths influence droplet dynamics, optimal patterns for water repellency may be identified, especially by minimizing the contact time and maximizing the restitution coefficient.
• Furthermore, the data could also be reused for machine learning and predictive modeling of droplet-surface interactions. By training AI models on the dataset, algorithms that predict the maximum spreading factor, restitution coefficient, and contact time for a given surface structure and liquid can be developed. These models could be used to optimize surface designs without the need for extensive experimental testing, saving time and resources.

## 2 Background

Superhydrophobic surfaces exhibit extreme water repellency, characterized by a high contact angle (above 150°) and low contact angle hysteresis (below 10°). Their remarkable ability to repel impacting water droplets makes them highly potential for applications such as icing mitigation, self-cleaning, and water-repellency, where minimizing contact time between the surface and liquid is essential [1]. Upon impacting a superhydrophobic surface, a droplet undergoes a spreading phase, during which the droplet deforms into a disc shape as its kinetic energy transforms into surface, followed by a retraction phase, during which the capillary forces drive the liquid inwards, restoring the droplets initial shape. Finally, the droplet detaches and rebounds from the surface. The extent of rebound depends on several factors, including droplet size and impact velocity [2], surface morphology [3], and fluid thermophysical properties such as density, surface tension [4] and viscosity [5]. Understanding how these parameters influence the spreading, rebound, and contact time is crucial for research and development of water-repellent surfaces and optimizing their performance in self-cleaning and anti-icing applications. Moreover, insights into droplet dynamics are valuable in other fields, such as inkjet printing and biomedicine, where precise droplet control ensures better material deposition, improved coating uniformity, and enhanced efficiency in microfluidic and diagnostic applications.

## 3 Data Description

**Fig. 1:**
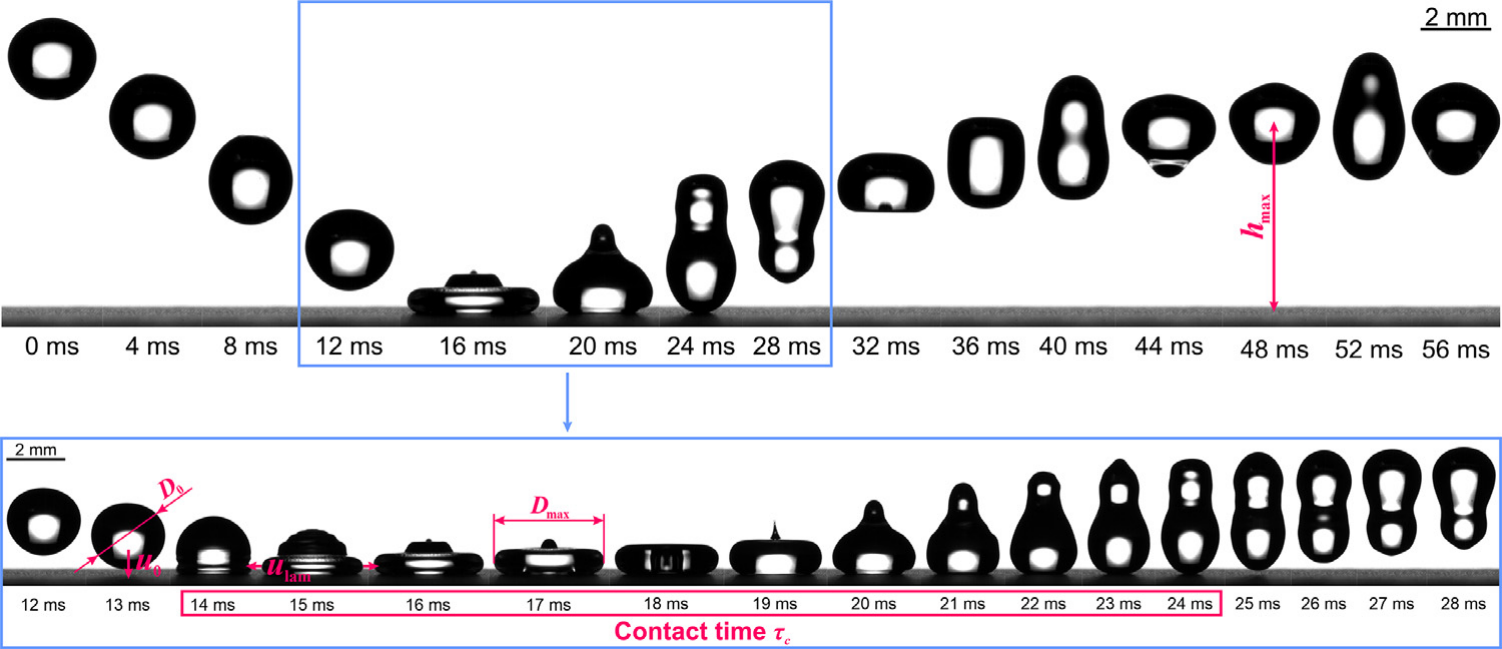
Key droplet-surface interaction parameters, shown on a series of snapshots for a sample recording.

The dataset [6] includes a quantitative description of droplet impact, spreading, and rebound on superhydrophobic surfaces with laser-made microchannels. Alongside droplet-side parameters (diameter, velocity, density, surface tension, and dynamic viscosity) and surface-side parameters (distance between adjacent laser beam passes and approximate average depth of laser-made channels), the following parameters were calculated and/or extracted via image processing. Firstly, the Weber and Reynolds dimensionless numbers are provided for the droplet. Then, the droplet-surface contact time, energy efficiency of the rebound, droplet maximum spreading factor, and maximum lamella velocity during the spreading phase are given. The main parameters describing the droplet-surface interaction that were extracted from the high-speed footage, are shown on snapshots from one of the recordings in Fig. 1.

**Fig. 2:**
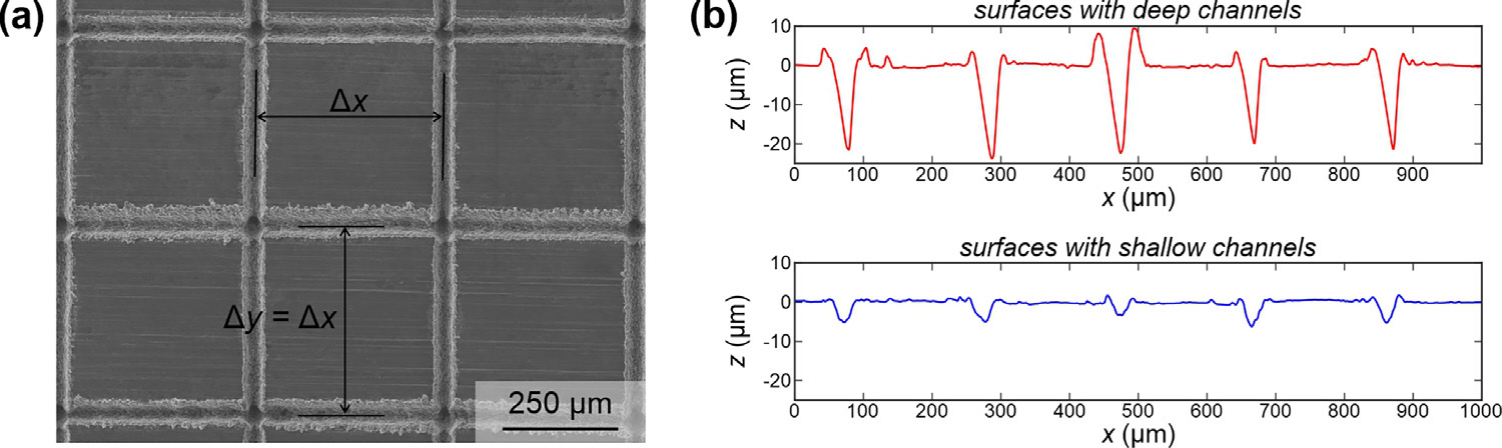
An example of an SEM image with depicted cell spacing value (a) and examples of surface profiles for surfaces with deep (top) or shallow (bottom) channels (b).

The main dataset is supplemented by another dataset providing the relevant droplet impact and spreading parameters for droplet impacts on a smooth hydrophobic surface. Additionally, contact angles are provided in three separate files, and SEM images of all tested surfaces are provided in a single folder. An example of an SEM image is shown in Fig. 2(a) including the dimensions of the cell spacing (Δx=Δy), while Fig. 2(b) shows an example of the deep surfaces profile (top) and shallow surface profile (bottom) recorded by a contact profilometer.

The main dataset (file “Rebound Data.csv”) consists of 1498 water-glycerol droplet impact experiments on monolayer-functionalized laser-structured aluminum samples organized in a single table. Each row represents a single measurement and includes the following parameters (listed as columns from left to right):

a) sample name,
b) droplet diameter (in m),
c) droplet impact velocity (in m s^−1^),
d) density of the droplet (in kg m^−3^),
e) surface tension of the droplet (in N m^−1^),
f) dynamic viscosity of the droplet (in Pa·s),
g) distance between adjacent laser beam passes (in µm),
h) approximate average depth of laser-made channels (in µm),
i) Reynolds number (dimensionless),
j) Weber number (dimensionless),
k) droplet-surface contact time (in seconds),
l) energy efficiency of the rebound (dimensionless),
m) droplet maximum spreading factor (dimensionless), and
n) maximum lamella velocity during the spreading phase (in m s^−1^).

The supplementary dataset (file “Rebound Data - REF-H.csv”) consists of 125 water-glycerol droplet impact experiments on a monolayer-functionalized smooth, hydrophobic aluminum sample organized in a single table. Each row represents a single measurement and includes the following parameters (listed as columns from left to right):

a) sample name,
b) droplet diameter (in m),
c) droplet impact velocity (in m s^−1^),
d) density of the droplet (in kg m^−3^),
e) surface tension of the droplet (in N m^−1^),
f) dynamic viscosity of the droplet (in Pa·s),
g) Reynolds number (dimensionless),
h) Weber number (dimensionless),
i) droplet maximum spreading factor (dimensionless), and
j) maximum lamella velocity during the spreading phase (in m s^−1^).

The supplementary dataset providing water contact angles (file “Contact angles - Water.csv”) is organized in a single table, where the columns list sample names (from D50 on the far left to S800 on the far right) and the following wettability metrics as rows (from top to bottom):

a) static contact angle (water droplets),
b) advancing contact angle (water droplets),
c) receding contact angle (water droplets),
d) contact angle hysteresis (water droplets).

The supplementary dataset providing contact angles of the highest concentration of the water-glycerol mixture used (91 wt.%) (file “Contact angles - Water-Glycerol_91wtPerc.csv”) is organized in a single table, where the columns list sample names (from D50 on the far left to S800 on the far right) and the single row lists the static contact angle for each of the samples.

The supplementary dataset providing advancing contact angles for water-glycerol mixtures on selected surfaces (file “Advancing contact angles - Water-glycerol mixtures.csv”) is organized in a single table, where the rows list sample names (namely, D50, D200, D800, S50, S200, and S800 in the provided order) and the advancing contact angles for the following water-glycerol mixtures, provided as columns (from left to right):

a) 20 wt.%,
b) 60 wt.%,
c) 78 wt.%,
d) 91 wt.%.

The supplementary folder “SEM images of the test surfaces” provides SEM images of all 12 laser-textured surfaces and of the smooth, hydrophobized surface at three selected magnifications (39 images in total, .tif format). The name of each image provides the sample name and the magnification of the image. For example, image “D50_43.tif” depicts the D50 surface at 43 × magnification.

## 4 Experimental Design, Materials and Methods

### 4.1 Samples

Aluminum alloy (1050A H24, ≥ 99.5 % Al) plates with a thickness of 1.0 mm and a square shape (38 × 38 mm) were used as substrates in this study. The substrates underwent functionalization with no polishing or other preparation steps. Prior to subsequent surface treatment, the substrates were wiped down with acetone and a lint-free wipe to remove any remnants of the adhesive as one side was originally covered with a protective polymer foil. Then, the substrates were ultrasonicated in isopropyl alcohol (IPA) for 10 min to remove any other contaminants, followed by drying in ambient air.

### 4.2 Surface treatment

To produce superhydrophobic surfaces on the aluminum substrates, laser texturing was combined with hydrophobization. First, the samples were laser-textured using a nanosecond fiber laser system (FL Mark-C with JPT Opto-electronics "M7 30 W" MOPA source). Deep channels (26.1±3.14 µm deep) were fabricated with a scanning speed of 400 mm s^−1^, pulse frequency of 80 kHz, pulse length of 100 ns, and pulse fluence of 45.8 J cm^−2^. To produce shallow channels (6.0±0.70 µm deep), the values were changed to 1650 mm s^−1^, 110 kHz, 45 ns, and 33.3 J cm^−2^. A square scanning grid with cell spacing values of {50, 100, 200, 400, 600, 800} µm was used. The name of each surface is composed of the channel depth (D = deep, S = shallow) and the spacing value in microns.

Following the laser texturing, the samples were again sonicated in IPA to remove loosely adhered particles and powder, followed by drying in ambient air. After drying, the samples were exposed to UV/ozone cleaning for 10 min to prepare the surface for hydrophobization. Immediately after UV/ozone cleaning, each surface was hydrophobized by applying several drops of a 3 mM IPA solution of 1H,1H',2H,2H'-perfluorododecil-1-phosphonic acid (FDPA) to the surface to completely cover it with the solution. The surfaces were left to dry under ambient conditions for 15 min and were then transferred in a convection oven where drying was finished for 1 h at 120 °C, which also helped strengthen the bond between the surface and the hydrophobic agent. The hydrophobization process produces a self-assembled monolayer on the surface with a thickness in the nanometric range.

### 4.3 Surface characterization

Static contact angles were measured using a goniometer (Ossila) by depositing droplets onto the surfaces and observing the resulting contact angle. Each measurement was repeated 5 times. Static contact angles were recorded using water and the most concentrated water-glycerol mixture (91 wt.% glycerol content). Furthermore, static contact angles were also measured at intermediate water-glycerol concentrations on select surfaces to verify the trends. Processing of the videos was performed in Ossila’s proprietary software.

Advancing contact angles were measured using the droplet inflation/deflation method on a custom-made experimental setup for goniometry equipped with a CMOS camera and a strong LED backlight. A syringe pump was used to inflate the droplets while the movement of the triple contact line across the surface was recorded with the camera. Processing of the videos was performed using custom-developed algorithms in MathWorks MATLAB environment.

SEM imaging was performed using a JCM-7000 NeoScope SEM, utilizing the secondary electron detector and operating at an accelerating voltage of 15 kV.

### 4.4 Experimental setup

**Fig. 3:**
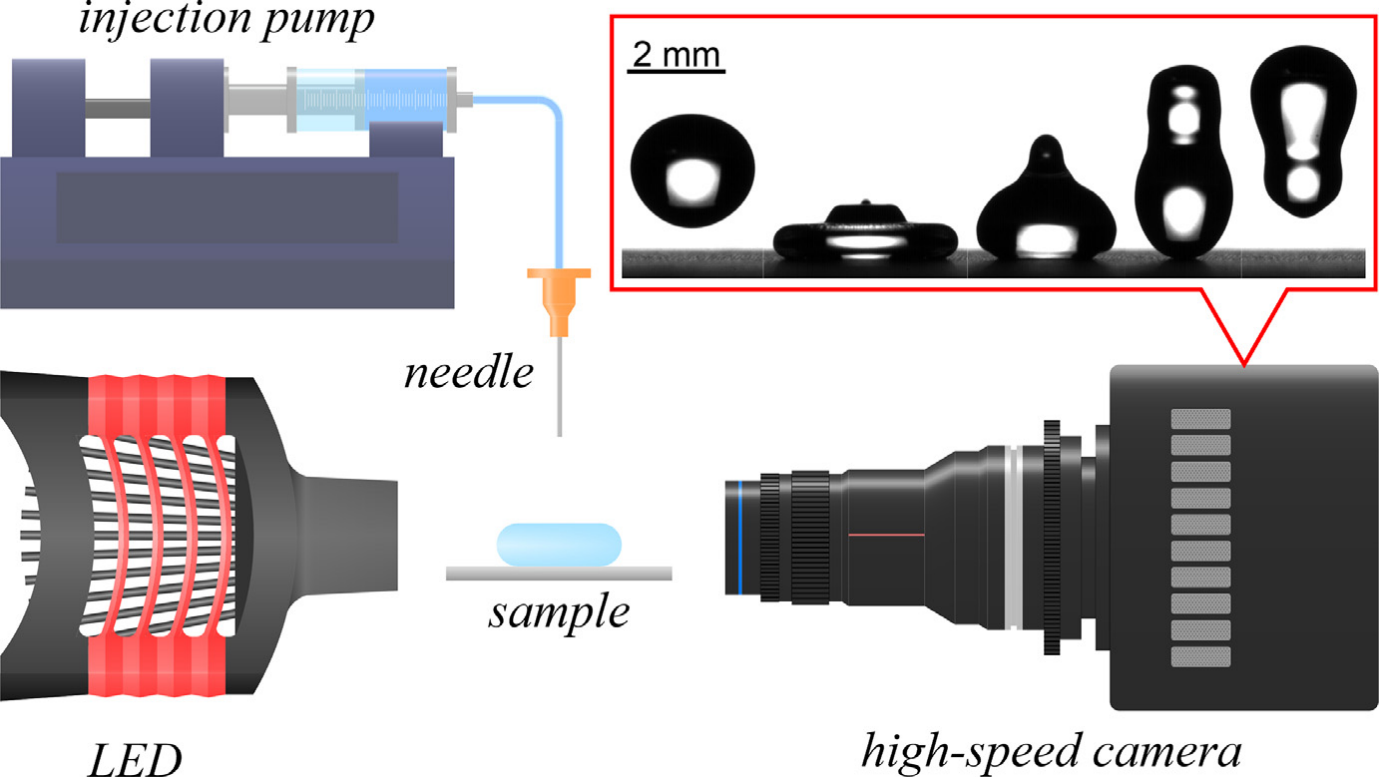
Schematic of the experimental setup for droplet impact evaluation.

Droplet impact was evaluated using a custom-made experimental setup shown in Fig. 3. Droplets of controlled size were produced using a syringe pump (Harvard Apparatus PHD 2000) and a blunt G27 needle. To vary the droplet impact velocity, the needle was mounted onto a 3D-printed holder with adjustable height. A two-axis linear translation stage was used for precise positioning of the sample beneath the needle. Droplet impact was captured using a high-speed optical camera (Photron FASTCAM Mini UX100) equipped with a macro lens (Laowa 100mm f/2.8 2x Ultra Macro APO), achieving a spatial resolution of ∼12 µm px^−1^. A white LED backlight (Thorlabs SOLIS-2C powered by a DC2200 controller) was used for illumination. The temperature and relative humidity were monitored during measurements.

### 4.5 Measurement protocol

Droplet impact measurements were conducted using water-glycerol binary mixtures with 0, 20, 60, 78 and 91 wt.% glycerol, spanning the viscosity from 1 to 160 mPa s. The droplets were generated by infusing the mixtures through a needle at a constant flow rate of 150 µL min^−1^. The droplet volume ranged from 6 and 8 µL, depending on the glycerol mass fraction; with a standard deviation of 0.4 µL for droplets of the same composition. The needle was set to five different heights, varying the impact velocity from 0.5 to 1.6 m s^−1^. The impact was captured with a high-speed camera at 5000 fps. The measurements were repeated five times for each combination of the surface, height and solution concentration. Prior to each measurement, the temperature was recorded. Post-processing of the captured data was performed using custom-developed algorithms in the MathWorks MATLAB environment.

### 4.6 Data reduction

**Fig. 4:**
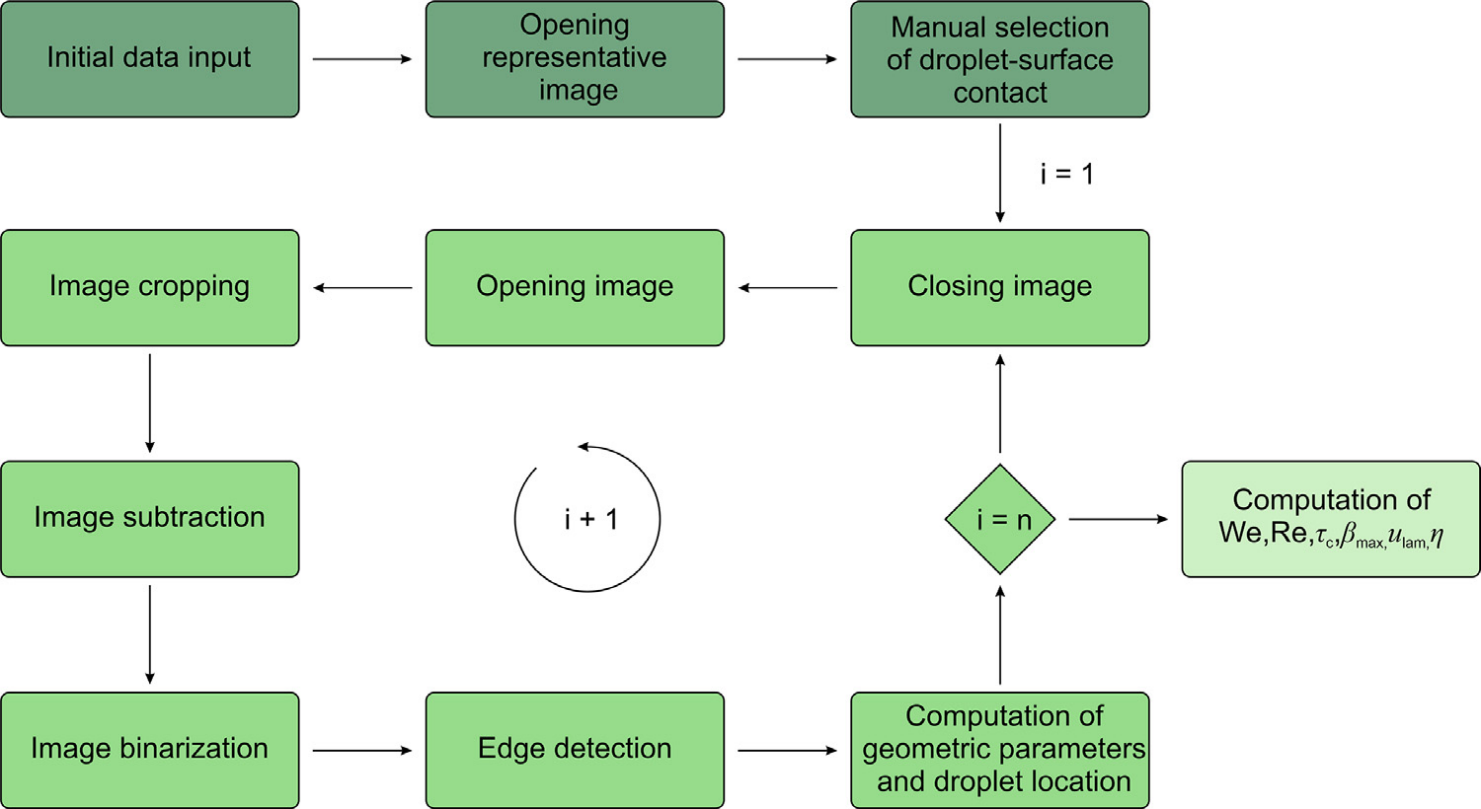
Flowchart of the image-processing routine for extracting the main parameters describing the droplet-surface interaction from the high-speed footage.

The captured footage of droplet rebound was processed using an in-house developed image-processing routine in MathWorks MATLAB R2024b, schematically illustrated in a flowchart shown in Fig. 4. Initially, the vertical coordinate of the aluminum surface was user-selected based on a representative image of droplet-surface contact where one can clearly distinguish the three-phase contact line. The original image was then cropped, removing the pixels under the user-defined point. Following this step, a background image without a droplet was utilized to isolate the droplet in each frame by means of image subtraction. Next, threshold-based binarization was adopted, where the region with the droplet was assigned 1 while the rest of the image was set to zero. Based on the identified boundary between both regions the droplet contour was obtained in each frame, enabling the computation of geometric parameters and droplet location. The moment of impact was defined as the frame where the droplet reaches the lowest *y* coordinate (*i.e.*, when the bottom extremity of the region of 1s came to the bottom edge in the image). Immediately prior to this frame, the velocity and volume of the droplet were recorded, and the Weber and Reynolds number were calculated:

(1) $$\text{We}=\frac{\rho u_{0}^{2}D_{0}}{\gamma }$$

(2) $$\text{Re}=\frac{\rho u_{0}D_{0}}{\mu }$$

where *ρ, γ* and *μ* are the density, surface tension and dynamic viscosity of the liquid, *u*_0_ is the droplet impact velocity, and *D*_0_ is the initial droplet diameter. During the spreading phase of the impact, the droplet radial extension in both directions was recorded and used to compute the spreading coefficient *β*, while the maximum speed of both extremities determined the lamella velocity *u*_lam_. In this way, *β* is defined as the ratio between the droplet’s horizontal extension and the initial droplet diameter prior to impact. The maximum value was reached at the transition between the spreading and retracting stages of rebound and was denoted as *β*_max_. After retraction, the droplet detached from the surface, which marked the end of the first rebound event. The length of droplet rebound is described by the contact time *τ*_c_, providing the temporal difference between the frame of first contact and the frame when the droplet region was separated from the bottom image edge (*i.e.*, the array of 1s is separated from the *y = 0* coordinate by at least one zero value). Following rebound, the droplet’s center of gravity began to vertically rise until a maximum value, at which point the potential energy was calculated relative to the sample’s surface. The energy “efficiency” of the rebound *η*_reb_ was calculated as the ratio of potential energy in the droplet’s highest point of rebound to the kinetic energy of the droplet immediately before the impact:

(3) $$\eta_{\text{reb}}=\frac{E_{after\;rebound}}{E_{before\;rebound}}=\frac{E_{potential}}{E_{kinetic}}\;$$

The restitution coefficient *ε*, which represents the ratio between the droplet velocity after the rebound and before it, can be calculated from the energy efficiency of the rebound as:

(4) $$\varepsilon =\sqrt{\eta_{reb}}\;.$$

Temperature-dependent thermophysical properties of a given binary mixture (*i.e.*, density, dynamic viscosity, and surface tension) were determined from [7], based on the measured temperature of the liquid before the experiments with a given water-glycerol mixture on each surface. Second-degree polynomials were used for surface tension and density, while an exponential function was utilized to describe the changes in dynamic viscosity. These functions were obtained by fitting the data by Takamura et al. [7] for various temperatures; the referenced study utilized identical concentrations of water-glycerol mixtures as featured in the present dataset.

Surface tension of the droplet was calculated by inserting the droplet temperature (in°C) into the following equation, yielding a result in mN m^−1^. Table 1 provides the values of coefficients for water-glycerol mixtures of different concentrations.

(5) $$\sigma \left(T\right)=a_{2}T^{2}+a_{1}T+a_{0}\;.$$

**Table 1:** Water-glycerol binary mixture surface tension approximation coefficients.

Glycerol wt.%	*a*_2_	*a*_1_	*a*_0_
0	−2.9819e-17	−0.1460	76.1100
20	−0.0017	−0.0105	72.6050
60	7.5000e-4	−0.1595	71.3950
78	−1.0000e-3	−0.0300	68.4000
91	2.5000e-4	−0.1205	68.8050

Density of the droplet was calculated by inserting the droplet temperature (in°C) into the following equation, yielding a result in kg m^−3^. Table 2 provides the values of coefficients for water-glycerol mixtures of different concentrations.

(6) $$\rho \left(T\right)=b_{2}T^{2}+b_{1}T+b_{0}\;.$$

**Table 2:** Water-glycerol binary mixture density approximation coefficients.

Glycerol wt.%	*b*_2_	*b*_1_	*b*_0_
0	−0.0075	0.1950	997.05
20	−0.0050	−0.0500	1050.00
60	5.8549e-16	−0.5400	1166.90
78	0.0150	−1.4700	1227.70
91	0.0125	−1.3050	1256.55

Dynamic viscosity of the droplet was calculated by inserting the droplet temperature (in°C) into the following equation, yielding a result in mPa·s. Table 3 provides the values of coefficients for water-glycerol mixtures of different concentrations.

(7) μ(T)=cexp(dT).

**Table 3:** Water-glycerol binary mixture dynamic viscosity approximation coefficients.

Glycerol wt.%	*c*	*d*
0	1.4780	−0.0179
20	2.7485	−0.0205
60	22.2591	−0.0330
78	113.8909	−0.0429
91	662.1278	−0.0551

## Limitations

None.

## Ethics Statement

All authors have read and followed the ethical requirements for publication in *Data in Brief* and are confirming that the current work does not involve human subjects, animal experiments, or any data collected from social media platforms.

## CRediT Author Statement

**Matic Može:** Conceptualization, Methodology, Formal analysis, Resources, Writing - Original Draft. **Samo Jereb:** Methodology, Software, Formal analysis, Data Curation, Writing - Original Draft, Visualization. **Robert Lovšin:** Methodology, Formal analysis, Investigation. **Jure Berce:** Software, Validation, Formal analysis, Data Curation, Writing - Review & Editing. **Matevž Zupančič:** Software, Validation, Resources, Writing - Review & Editing, Project administration. **Iztok Golobič:** Resources, Project administration, Funding acquisition.

## Data Availability

Mendeley DataDataset on droplet spreading and rebound behavior of water and viscous water-glycerol mixtures on superhydrophobic surfaces with laser-made channels (Original data)
